# Controlling unequal surface energy results caused by test liquids: the case of UV/O3 Treated PET

**DOI:** 10.1038/s41598-022-10816-6

**Published:** 2022-04-26

**Authors:** Bilge Nazli Altay, Paul D. Fleming, Md Arifur Rahman, Alexandra Pekarovicova, Bruce Myers, Cem Aydemir, Arif Karademir

**Affiliations:** 1 College of Engineering Technology, Print and Graphic Media Science, Rochester Institute of Technology, Rochester, NY 14623-5608 USA; 2 Chemical and Paper Engineering, Western Michigan University, Kalamazoo, MI 49008-5462 USA; 3 Institute of Pure and Applied Sciences, Printing Technologies, Marmara University, 34722 Istanbul, Turkey; 4 Thermoplastic Polyurethane Research, BASF Corporation, 1609 Biddle Ave., Wyandotte, MI 48192 USA; 5 Faculty of Forestry, Forestry Industry Engineering, Division of Pulp and Paper, Bursa Technical University, 16310 Bursa, Turkey

**Keywords:** Chemistry, Energy science and technology, Engineering, Materials science, Mathematics and computing, Nanoscience and technology, Physics

## Abstract

Ultraviolet/ozone (UV/O_3_) treatment has been reported to be an effective method to modify properties such as wettability, adhesion or adsorption of plastic surfaces. The change in the surface is measured by contact angle analysis, which employs liquids and their surface tensions (ST) to estimate the surface energy (SE). We found two different practices in the scientific community: (1) the majority of researchers adopted the ST value of liquids from the literature, while (2) other researchers conducted real-time measurements in the lab under ambient conditions prior to SE estimation. To the best of our knowledge, there is no study that compares the difference between the two practices. One study was found to show different SE methods generating unequal SE values for the same substrate. However, there was no definitive conclusion backed by general thermodynamics rules. In this study, we presented (1) a statistical significance test that showed the literature and experimental ST values are significantly different, and studied (2) the effect of different liquid pairs on the SE estimation for UV/O_3_ treated poly(ethylene terephthalate) (PET) substrate. Modification techniques such as atmospheric pressure plasma or chemical modification were studied previously to examine PET’s wettability and the SE. The UV/O_3_ treatment was studied to improve adhesion and to modify its chemical properties for adsorption. In contrast, we studied (3) the effect of UV/O_3_ on wettability at different timeframes and addressed (4) how to control unequal SE based on a method that was refined on a rigorous thermodynamic three-phase system. It must be noted that this method can be generalized to other types of solid surfaces to estimate thermodynamically self-consistent SE values. This work also provides (5) a web-based calculator that complements computational findings available to the readership in the data availability section.

## Introduction

Plastic substrates are mainstream materials to fabricate a wide range of applications, including printed and flexible electronics, biomedical devices and packaging^1–3^. The relation between the surface characteristics of plastics and the ST of dispersions plays an important role in the leveling, film formation and adhesion behavior of dispersions such as inks, coatings and adhesives. Surface characteristics, such as hydrophilicity, morphology, wettability and roughness, affect the homogeneity of dispersion films and thus the final properties of the intended applications^4,5^. PET became a viable substrate not only for packaging applications but also for printed electronics due to its superior strength and resilience, high melting points, tensile strength, good impact resistance, outstanding processability and considerable cost advantage over the other plastic options^6–8^. The dimensional stability of PET can be enhanced by a heat stabilization process at temperatures up to 150 °C^8^. In terms of packaging, PET exhibits glass-like transparency, low odor and gas–water permeability and is very suitable for processes such as hot embossing, lamination, molding and printing. The most important durability of PET opposed to other plastics is its chemical inertness. However, this property makes PET have poor wettability and requires surface treatment prior to processing in various industrial fields^1,7^. The contact angle method characterizes the effect of surface treatment, and inherent or changed wetting/dewetting behavior of materials, which can have a profound effect^9^. The ST and contact angle method quantify the SE value and is of use, especially if the dispersion is poor^4^. The SE value is the sum of polar and dispersive components and shows the wettability behavior of liquids between the two components affinity with a surface. The effect of surface treatment can also be measured using dyne pens and solutions in the production environment; however, the process is subjective and does not reveal the values of polar and disperse components. Increasing the polar component of substrates, and monitoring it, is especially important to achieve proper adhesive bonding in polymers^10^.

The prevalent strategy to optimize wettability, film formation and adhesion is to decrease the ST of dispersions and/or to increase the SE of substrates by gas-phase surface modification processes such as corona discharge, plasma treatment and flame treatment^4,10^. An alternative and more advantageous strategy is to establish a good correlation between dispersion ST and the Hansen solubility parameters of substrates, which ensures that liquid and surface molecules have the maximum chance of interacting^4^. Inaccurate SE measurement may make unreliable results and erroneous conclusions that lead to observe liquids forming droplets on the plastic surface or poor adhesion and bonding^10^.

To date, significant research has been published on SE characterization in chemistry^11,12^, coatings^13^, printing^14–16^, adhesives^17,18^, flexible electronics^19–22^, biomaterials^23,24^, oil recovery^25^ and medical engineering^26^. These publications covered the fundamentals of SE in relation to wettability, solubility, contamination, adsorption, absorptivity, adhesion, and bonding^27–30^. Numerous theoretical or semiempirical SE estimation models have been developed by Fowkes^31–33^, Owens–Wendt (OW)^34^–Rabel^35^–Kaelble^36^, van Oss et al.^37^, Fox^38,39^, Neumann et al.^40–42^, Wu^43,44^, Zisman^45^, Schultz^46^; however, the OW has been found to be the most commonly used method for SE characterization^47–61^. In the OW method, the ST of at least two liquids with known dispersive and polar components and the contact angles of the same liquids on a given surface are used to define the SE^62,63^. The common ST and contact angle measurement techniques are presented in Table 1^64,65^, followed by the liquids that are frequently used for the SE characterization in Table 2.

**Table 1:** Surface tension and contact angle measurement techniques.

Surface tension, static methods	Surface tension, dynamic methods	Contact angle methods
Pendant drop	Bubble pressure	Sessile drop
Wilhelmy plate	Drop volume/weight	Wilhelmy
Ring/Du-Noüy	Falling curtain (Mach angle)	Washburn
Spinning drop		Top-view distance
Sessile drop

**Table 2:** Commonly used test liquids for contact angle measurements.

Liquid	References
Water	^1,2,11,17,47–60,66,66^
Diiodomethane	^11,17,47–49,51–54,56,57,59,60^
Thiodiglycol	^48^
Ethylene glycol	^17,48–50,54,55,57,59,67^
Formamide	^17,50,58^
Propylene glycol	^17,50^
Glycerol	^17,55,58,67^
2-Ethanol amine	^55,58^
Hexadecane	^2,11,58^
Dimethyl sulfoxide	^17,67^
1,2,6-Trihydroxyhexane	^17^
Tricresyl phosphate	^17^
1-Bromonaphthalene	^17^

For SE characterization, the majority of the literature introduce the Young–Dupré equation^68,69^ (Eq. 1) as the core principle to estimate SE from the three interfacial tensions as follows:

1 $${\gamma }_{SV}= {\gamma }_{SL}+ {\gamma }_{LV}\,\mathrm{cos}\,\theta$$

where ${\gamma }_{SV}$ is the solid–vapor interfacial energy; ${\gamma }_{SL}$ is the solid–liquid interfacial energy; ${\gamma }_{LV}$ is the liquid–vapor interfacial energy; and $\theta$ is the contact angle between the tangent lines along the liquid–vapor interface and solid–liquid interfaces of the liquid drop. The formula in Eq. (1) contains two unknowns^17^, and the degree of contact angle corresponds to an SE level in the equilibrium system formed between the liquid and the solid on the condition that the surface is smooth, nonporous, nonsorptive, and homogeneous^34,70^; hence, the contact angle is unattainable since the model of an ideal solid surface is physically unrealizable^71,72^. This is why the aforementioned semiempirical methods became prevalent; however, they were found to estimate unequal SE values for the same surface, and the results were very dependent on the liquids employed^67^. It must also be noted that in the literature, two different practices were found, revealing that the majority of researchers adopted the ST value of the liquids directly from the literature^11,17,47–56,58^, while other researchers employed real-time measurements in the lab before SE characterization^11,17,22,63,73^. No study has been found that addresses either if any significant difference exists between these two practices or if any method demonstrates an improvement to control unequal SE estimations caused by the liquids employed.

In this study, we investigated the SE of UV/O_3_ treated PET at different timeframes by measuring contact angles using the sessile drop method. Unlike previous research, we determined the difference between the experimental ST of test liquids measured in the lab using the pendant drop method and the corresponding ST values reported in the literature. By pairing the liquids in different combinations, we analyzed the effect of liquids on SE estimations using the most common method of OW and the most recent method of Altay-Ma-Fleming (AMF)^11^. Contrary to prior studies, hypothesis tests were performed in each step using the *t*-test analysis to establish if any significant difference exists between the groups’ mean response, based on a 95% confidence limit.

## Materials and methods

### Surface tension, contact angle and UV/ozone treatment

The test liquids for the study were ultrafiltered deionized water (DI) (Fisher Scientific, Fair Lawn, NJ), diiodomethane (MI) (Sigma Aldrich, St. Louis, MO. 99% purity, 3.325 g/cc) and hexadecane (HD) (Sigma Aldrich, St. Louis, MO. 99% purity, 0.770 g/cc). First, the ST value of the liquids was surveyed in the databases and then measured 10 times using the pendant drop method under ambient conditions in the lab (Western Michigan University, Center for Printing and Coating Research) with an FTA 200 flexible video system and FTA 32 software (First Ten Angstrom, Portsmouth, VA). The shape of a pendant liquid drop under equilibrium conditions was described by the Laplace-Young equation^74,75^. Using FTA32 software, the hanging liquid drop was analyzed by the Bashforth-Adams technique to solve the Laplace-Young equation^76^. The PET surface was a Melinex ST506 (DuPont, Wilmington, DE) for the static contact angle study via sessile drop profile techniques on the same FTA system. The PET samples were cut into 0.5 × 6 in. pieces and mounted on a device holder with double-sided tape. Each test liquid was deposited onto the substrate under ambient conditions. The evolution of the contact angle changing with time was video recorded and plotted as a curve of contact angle vs. time. The average of three contact angle analysis was reported and used for the SE estimation. The angles were measured after the drop has been in contact with the substrate for 5 s according to TAPPI T-458 method. The UV/O_3_ treatment of PET was performed using a cleaning device (Jelight, Irvine, CA. 144AX cleaner) at room temperature for 1, 3, and 6 min. The treated surface was measured immediately after the treatment.

### Surface topography measurement

A MultiMode 8 Atomic Force Microscope with Nanoscope V Controller (Bruker Nano Surfaces) was used to measure surface roughness of PET. 100 µm × 100 µm scans were acquired in ContactMode™ (also called constant-force mode). 2D and 3D height images were plotted to show relative roughness. Surface roughness was then performed to determine average roughness (Ra).

### Surface energy estimations

The test liquids were paired as DI/MI, MI/HD, and DI/HD. The SE was estimated first with the average ST value of the liquids found in the literature, then the average of 10 replicates was conducted in the lab using the methods of OW based on a two-liquid component model and the AMF. The AMF method is based on the Girifalco and Good method^77^, which supplements the Young–Dupré equation, has a dimensionless interfacial interaction parameter and is in line with Antonow’s generalized thermodynamic inequality relating the three interfacial tensions in a three-phase equilibrium system^78–80^. The AMF method calculates an α parameter^11^ (Eq. 2) that satisfies the inequality of ${\gamma }_{SV}+ {\gamma }_{SL}-{\gamma }_{LV} >0$, where

2 $${\gamma }_{SV}= \frac{{\gamma }_{LV}\,{cos}^{2}(\theta /2)}{\alpha }$$

### Statistical analysis

The hypothesis test was performed using *t*-test analysis to compare the groups’ mean response if variances in the two sample groups were different. The significance level, *p*-value, was set to α = 0.05 (95% confidence limit). A *p*-value less than 0.05 indicates that a significant difference exists between the samples, while a value over 0.05 indicates that the difference is not significant. The analysis of variance (ANOVA) statistical method was analyzed with JMP Pro 16 software.

## Results and discussion

**Figure 1:**
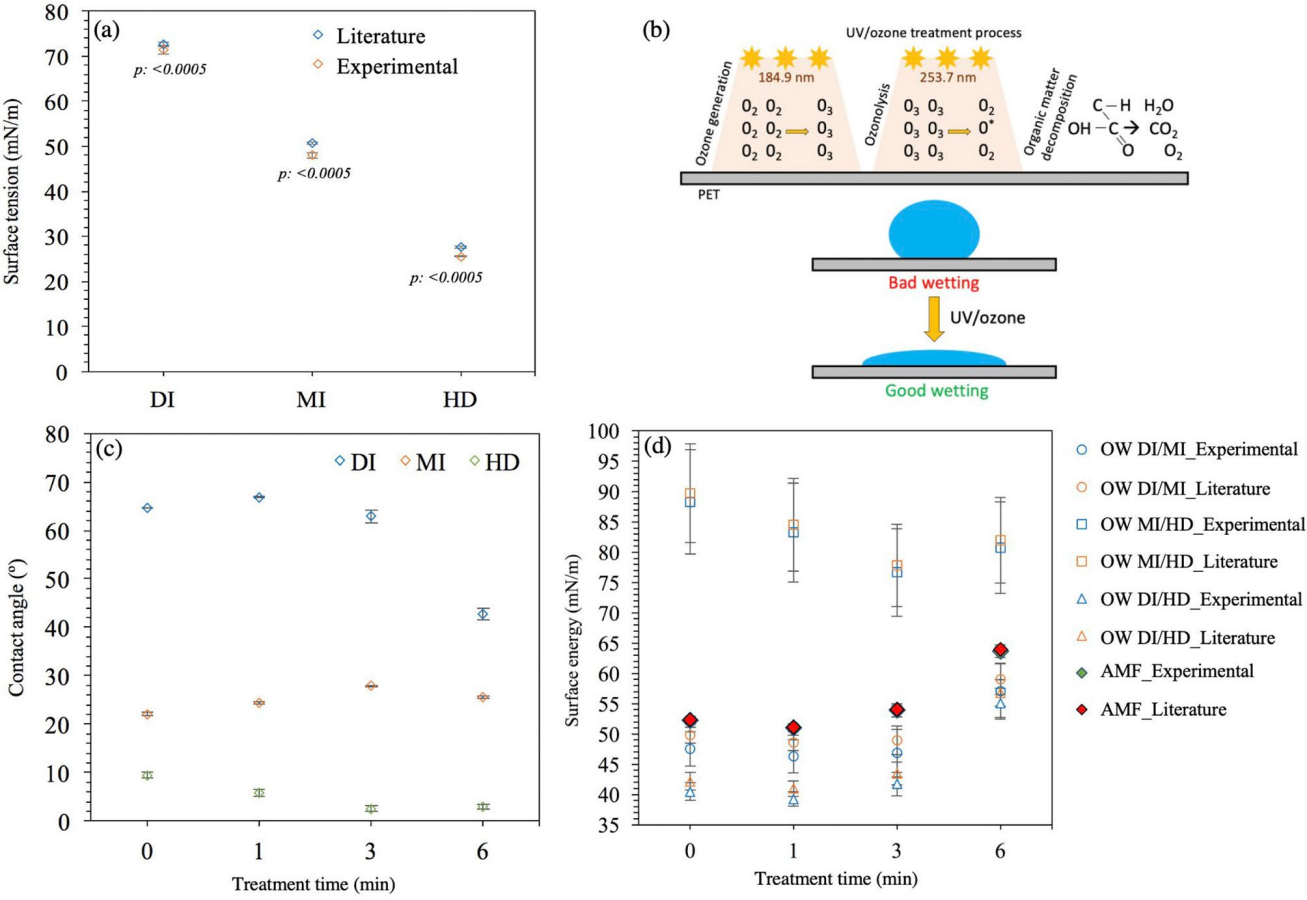
(**a**) Comparison of surface tension values found in the literature and experimented in the lab, (**b**) UV/ozone treatment process, (**c**) contact angles of test liquids on PET as a function of treatment time, (**d**) comparison of PET surface energy based on the OW and AMF.

The STs of three different liquids were obtained by surveying the literature (Table 3). The average STs determined for DI, MI and HD were 72.75 ± 0.25 mN/m, 50.72 ± 0.30 mN/m and 27.60 ± 0.29 mN/m, respectively (S1). The average of 10 experiments for the ST measurement in the lab was determined to be 71.36 ± 0.87 mN/m for DI, 48.03 ± 0.60 mN/m for MI, and 25.55 ± 0.11 mN/m for HD (S1). Statistical analysis calculated the *p*-values shown in Fig. 1a, indicating that the difference between the ST of test liquids in the literature vs. in the laboratory is significant^11,47–56,58^. The values measured in the lab were in line with the data reported previously^63^. The main reasons for obtaining lower values in lab experiments may be variation in the purity levels of chemicals, contamination, degradation of principle materials in the chemicals, temperature variations or ambient conditions. It may also be due to different optical resolutions, signal sensitivities, or numerical algorithms for drop shape evaluation of the device systems adopted or the methodology followed by the researchers^70,72^. The difference may be caused by some shorter chain hydrocarbons of some organic acids for HD^81^.

**Table 3:** Surface tension values from the literature of the selected liquids.

Liquid	Surface tension (mN/m)	References
DI	72.80	Harkins^82^
DI	72.80	DataPhysics^83^
DI	72.75 ± 0.36	Vargaftik^84^
DI	72.40	Amiri^85^
DI	72.74 ± 0.36	IAPWS^86^
DI	72.85 ± 0.10	Zdziennicka^87^
MI	50.82 ± 0.11	Zdziennicka^87^
MI	50.88	Körösi^88^
MI	50.00	Busscher^89^
MI	50.80	Ström^90^
MI	50.80	Parreidt^91^
MI	50.80	Dann^92^
HD	28.12	Jasper^93^
HD	27.64	Jasper^94^
HD	27.47	Rolo^95^
HD	27.42	Koefoed^96^
HD	27.50	van Oss^97^

**Figure 2:**
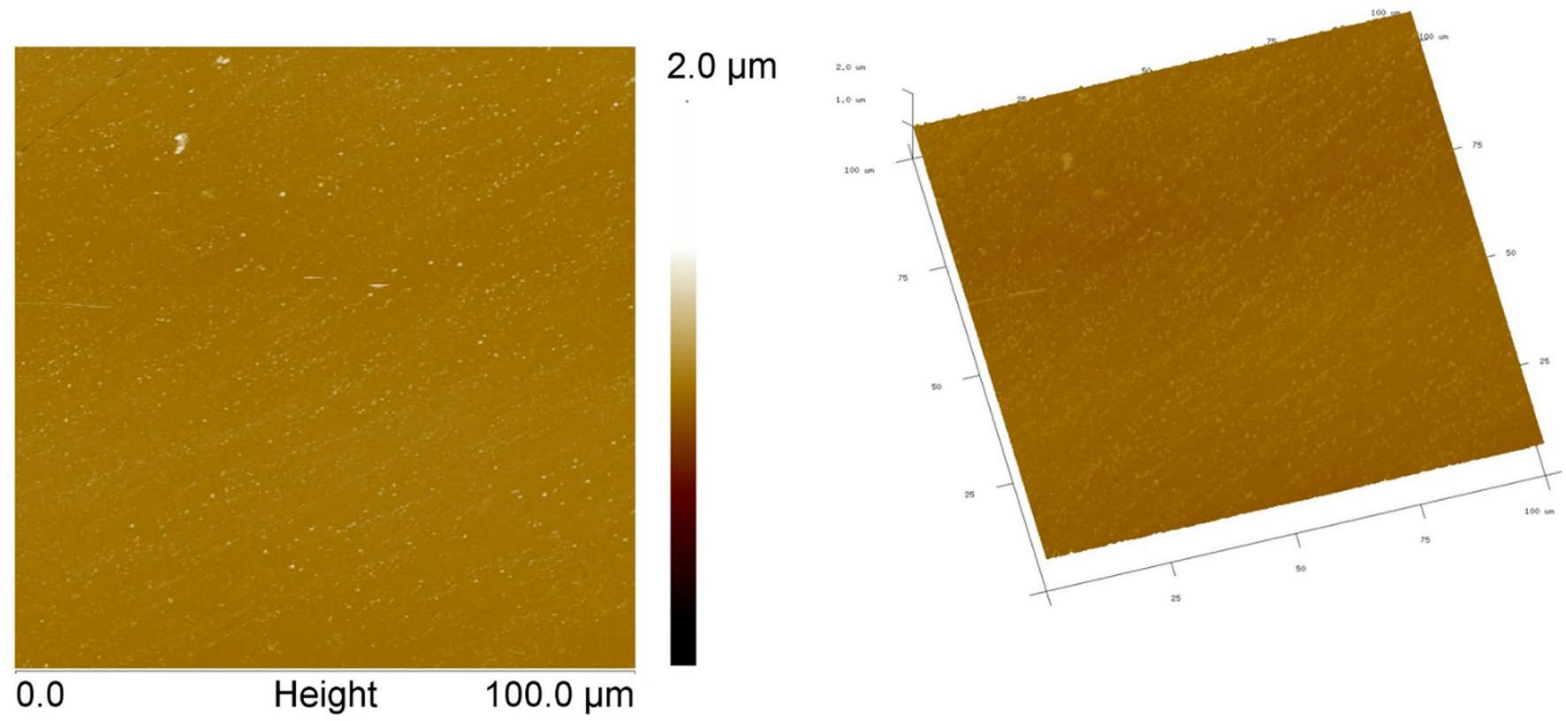
ContactMode™ Height images at 100 µm × 100 µm scan area of PET SBS substrate. 2-D (left) and 3-D (right) views. Z-scale for Height images is 2.0 µm. Tilt = 30°, Rotation = 15°.

Most plastic films are inherently low SE materials that repel the liquids. Instead of the liquids wetting out on the film surface, they bead up. The hydrocarbon contamination on the surface as a result of migrating additives or solvent residues and skin oils limits the bonding areas. The UV/O_3_ cleaning system is a photosensitized oxidation process that dissociates the aforementioned hydrocarbon contaminations by generating broadband UV radiation (Fig. 1b). The low-pressure mercury vapor grid lamp in the cleaning system generates two main wavelengths, one at 184.9 nm and one at 253.7 nm^16^. Atomic oxygen is generated when O_2_ is dissociated by 184.9 nm and O_3_ by 253.7 nm. The radiation of 253.7 nm is absorbed by most hydrocarbons, so the products of this excitation react with atomic oxygen to form simpler, volatile molecules to finalize the cleaning and surface modification process, thus improving wettability. Contact angles respond to any change in surface chemistry and changes in surface topography^16^. It is reported that the topographical features having dimensions of less than 100 nm does not significantly affect contact angle measurements and need be attributed to the changes in the surface chemistry of the treated polymers^16^. Using the AFM, the average roughness (Ra) of PET was measured as 12 ± 3 nm (Fig. 2).

Figure 1c depicts that the highest contact angle values were formed by DI, followed by MI and HD at all the treatment levels. Table 4 present the average contact angle values of all liquids at 0, 1, 3 and 6 min of treatment levels. The *p*-values from the *t*-test analysis showed no significant difference between the DI contact angles except for 6 min (S2a). The letter displays method also used in Table 4 to report of all pairwise comparisons in the connecting letters report column. Both SE and ST values are the sum of polar (hydrogen bond) and dispersive (non-polar) components; thus, increasing one component lowers the other^64^. DI has the highest ST (72 mN/m) and the highest polar fraction (51 mN/m) due to the hydrogen bonding in water molecules (21 mN/m dispersive). The lowest DI contact angle represents the highest wetting achieved at 6 min and indicates that longer treatment times increase polar fraction of PET. Oxidizing the aliphatic hydrocarbons generates an oxide layer on the surface that makes the PET more hydrophilic, thereby improve its wettability^3,98^. The slight differences between the DI angles at 0, 1 and 3 min may be caused by the dissolution of low-molecular-weight oxidized materials (LMWOM), which alters the localized ST of the DI when measured in air^16^ and leads to forming different contact angles. The mechanisms of UV/O_3_ treatment, the oxygen uptake of PET as an oxygen-containing polymer and the surface-oxidation treatment methods generating a water-soluble surface consisting of LMWOM have been discussed in another publication^16^.

**Table 4:**
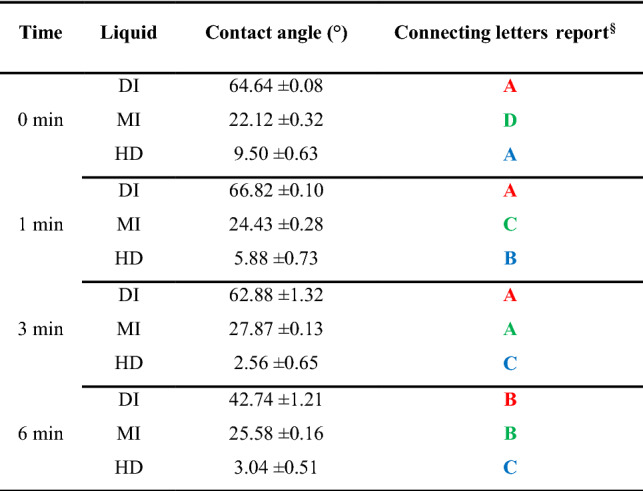
Contact angle at different UV/O_3_ treatment timeframes.

^§^Same color levels not connected by same letter are significantly different. ABCD lists the values from highest to lowest.

Plastic substrates exclusively form dispersive interactions, thus non-polar (dispersive) liquids easily wets the surface^64^. Both MI (*total ST: 50 mN/m dispersive*) and HD (*total ST: 27 mN/m dispersive*) are non-polar liquids; therefore, the overall contact angles of MI and HD formed on PET were significantly less than the DI. The MI contact angle slightly increased at 1 and 3 min of treatment time and then decreased at 6 min (Table 4, S2b). Since the UV/O_3_ treatment increases polar fraction of PET, the highest wetting for the MI was observed when there was no treatment. For the HD, the contact angle decreased at 1 and 3 min; however, the change was not significant between the 3 and 6 min (Table 4, S2c). The different contact angle formation for the non-polar liquids may be from the changes taking place in PET during UV light absorption, including the formation of carboxylic acid end-groups, terminal vinyl groups, phenols and the evolution of CO and CO_2_^16^. Since wide varieties of functional groups are known to form during surface treatment on the PET and known to be complex, the data suggest that the reaction between the HD and the relative functional group reaches saturation after 3 min and presents no further reduction in contact angle for^16,27,99^. The literature also reports that the SE of the water-soluble surface, consisting of LMWOM formed during surface treatment may be different than the insoluble underlying material (PET), causing difficulties for the interpretation of the angle data^16^.

**Figure 3:**
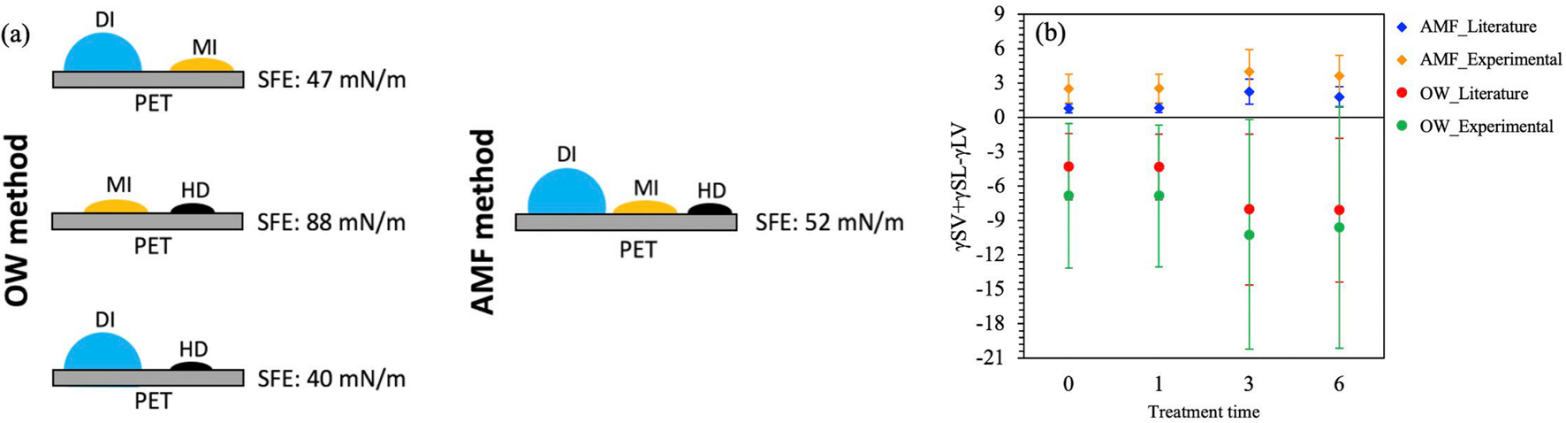
(**a**) The effect of test liquids on the SE analysis based on the OW and AMF methods, (**b**) Uncertainty of OW and AMF satisfaction of Antonow’s inequality.

The effect of liquids employed for the SE analysis was studied by pairing them as DI/MI, MI/HD, and DI/HD based on the OW method requiring ST of at least two liquids with known dispersive and polar components and the contact angles of the same liquids on a given surface^34,62,63^. Table 5 shows that the untreated PET SE estimated as 88.32 ± 8.63 mN/m with the MI/HD pair, 47.54 ± 2.85 mN/m with the DI/MI pair and 40.49 ± 1.45 mN/m with the DI/HD pair when the experimental ST values were used based on OW method (Fig. 1d, Fig. 3a). The OW estimated the highest value from the MI/HD non-polar liquid pairs relative to the DI/MI and DI/HD pairs. When the literature ST values were used, the SE was found to be 89.72 ± 8.10 with MI/HD pair, 49.80 ± 1.32 with DI/MI pair and 42.20 ± 1.50 mN/m for DI/HD pair (Fig. 1d). Similar results were observed at 1, 3 and 6 min of treatment levels (Table 5). The effect of liquid pairs on SE was found to be significant (S3a). The results showed that selecting different liquid pairs produces unequal SE for the same surface. On the other hand, the difference between the SE values of literature vs. experimental is found to be significant for the DI/HD and MI/HD pairs except the DI/MI pair (S3b). The high variation in the SE results indicates that the OW method is limited when different liquid pairs are used for the SE estimation, especially with the non-polar liquid pairs.

**Table 5:** SE estimations based on OW method at different UV/O_3_ treatment timeframes.

Time (min)	Literature			Experimental		
	DI/MI	MI/HD	DI/HD	DI/MI	MI/HD	DI/HD
0	49.80 ± 1.32	89.72 ± 8.10	42.20 ± 1.50	47.54 ± 2.85	88.32 ± 8.63	40.49 ± 1.45
1	48.54 ± 1.29	84.55 ± 7.64	40.99 ± 1.25	46.30 ± 2.76	83.27 ± 8.13	39.25 ± 1.19
3	48.94 ± 2.35	77.81 ± 6.81	43.48 ± 1.93	46.83 ± 3.91	76.66 ± 7.23	41.75 ± 1.93
6	59.04 ± 2.54	81.99 ± 7.11	56.80 ± 2.14	57.10 ± 4.61	80.76 ± 7.58	55.11 ± 2.39

The SE based on the AMF method are presented in Table 6. For the untreated PET, the SE was found to be 52.34 ± 0.21 mN/m and 52.22 ± 0.66 mN/m with the DI literature and experimental ST values, respectively. Equivalent SE values were observed for DI at each treatment level using the literature and experimental ST values, which suggests that the AMF method is insensitive to the variation in the ST (Table 6, Fig. 1d). According to AMF method, the liquids were used separately to estimate the SE. The liquid that estimates the highest SE (indicated with * in Table 6) provides an absolute lower bound and accepted to be the most accurate, meaning that the SE cannot be less than the absolute lower bound value based on the Antonow’s thermodynamics rule. In our study, the highest SE estimated by the DI at each treatment level. However, DI is not necessarily the liquid that provides the highest SE for all solid surfaces. The more the liquids used for the estimation, the more accurate lower bound can be estimated^11^. The highest value depends on the interaction between the liquid and the chemistry of the surface. Six cases are reported where the highest SE was provided by MI^11^.

**Table 6:** SE estimations based on AMF method at different UV/O_3_ treatment timeframes.

Time	Liquid	Literature	Experimental
0	DI	52.34 ± 0.21*	52.22 ± 0.66*
	MI	49.36 ± 0.32	47.22 ± 0.62
	HD	27.91 ± 0.31	25.56 ± 0.12
1	DI	51.09 ± 0.21*	50.99 ± 0.66*
	MI	48.95 ± 0.32	46.83 ± 0.61
	HD	28.02 ± 0.30	25.67 ± 0.12
3	DI	54.07 ± 0.63*	53.94 ± 1.10*
	MI	48.25 ± 0.30	46.17 ± 0.59
	HD	28.07 ± 0.30	25.71 ± 0.11
6	DI	63.98 ± 0.50*	63.69 ± 1.06*
	MI	48.71 ± 0.30	46.61 ± 0.60
	HD	28.06 ± 0.30	25.70 ± 0.11

Based on Antonow’s thermodynamics rule, the inequality difference must be positive^80^. The results show in Fig. 3b that the AMF satisfies the inequality (γ_SV_ + γ_SL_ − γ_LV_ > 0); however, all the OW values show violations at each treatment level (Experimental). The error bars for the AMF differences are relatively low, while in the corresponding OW values, the error bars are large and significantly negative throughout the range of the measurements. Similar behavior was reported for different substrates^11^.

## Conclusion

UV/O_3_ treatment was applied to investigate the wettability of PET at different timeframes. The effect was quantified by SE analysis via contact angle and ST measurements using three common test liquids: DI, MI and HD. Despite the prevalent practice of adopting ST of liquids established by fundamental research in a controlled environment, we measured ST in the lab under ambient conditions. The experimental ST results were compared to the literature values and found to be significantly different.

The contact angle measurement was used to observe wettability and the effect of liquids on SE. The highest wettability was found to be at 6 min of treatment and the contact angle of liquids was found to decrease for DI and HD but not MI. The liquids were paired in different combinations. Each liquid pair generated substantially different SE values for the same PET surface. It was observed that the SE deviation ranged from 25 mN/m to 50 mN/n depending on the liquid pair based on the OW theory and it is not clear how to decide which liquid estimates the accurate SE. The uncertainties of the method were found large and violated the general thermodynamic inequality for a three-phase equilibrium system. Based on the AMF method, the SE was estimated to be 52 mN/m for the untreated PET and 64 mN/m after the 6 min of treatment. Based on the test liquids, the SE varied 23–36 mN/m; however, the method is capable to point out the accurate SE. The method is found to be reliable to control unequal SE caused by the test liquids due to being refined on a rigorous thermodynamic three-phase system. The AMF method scales the dispersive and polar components of the total SE value based on the OW method; thus, further studies are needed for improvement.

## Supplementary Information

Supplementary Information.

## Data Availability

The datasets generated during and/or analysed during the current study are available from the corresponding author on reasonable request. The web-based AMF calculator can be reached by https://people.rit.edu/bnappr/AMF-Surface-Energy.html.
