# Supplementary material for: Controlling unequal surface energy results caused by test liquids: the case of UV/O3 Treated PET
Source: Sci Rep. 2022 Apr 26;12:6772. doi: 10.1038/s41598-022-10816-6 (PMC9043197; doi:10.1038/s41598-022-10816-6)
Supplement: Supplementary file 1 — Supplementary Information. [file 41598_2022_10816_MOESM1_ESM.docx]

**Controlling unequal surface energy results caused by test liquids: The case of UV/O3 Treated PET**

**Supporting Data**

**S1. Surface tension analysis: (Experimental (Lab), versus Literature**

Paired T-Test and CI: DI_Lab, DI_Literature - Descriptive Statistics

| Sample | N | Mean | StDev | SE Mean |
| --- | --- | --- | --- | --- |
| DI_Lab | 12 | 71.360 | 0.869 | 0.251 |
| DI_Literature | 12 | 72.752 | 0.252 | 0.073 |

Estimation for Paired Difference

| Mean | StDev | SE Mean | 95% CI for μ_difference |
| --- | --- | --- | --- |
| -1.392 | 0.891 | 0.257 | (-1.958, -0.826) |

*µ_difference: mean of (DI_Lab - DI_Literature)*

Test

| Null hypothesis | H₀: μ_difference = 0 |
| --- | --- |
| Alternative hypothesis | H₁: μ_difference ≠ 0 |

| T-Value | P-Value |
| --- | --- |
| -5.41 | 0.000 |

Paired T-Test and CI: MI_Lab, MI_Literature - Descriptive Statistics

| Sample | N | Mean | StDev | SE Mean |
| --- | --- | --- | --- | --- |
| MI_Lab | 10 | 48.025 | 0.595 | 0.188 |
| MI_Literature | 10 | 50.717 | 0.297 | 0.094 |

Estimation for Paired Difference

| Mean | StDev | SE Mean | 95% CI for μ_difference |
| --- | --- | --- | --- |
| -2.692 | 0.688 | 0.218 | (-3.184, -2.200) |

*µ_difference: mean of (MI_Lab - MI_Literature)*

Test

| Null hypothesis | H₀: μ_difference = 0 |
| --- | --- |
| Alternative hypothesis | H₁: μ_difference ≠ 0 |

| T-Value | P-Value |
| --- | --- |
| -12.37 | 0.000 |

Paired T-Test and CI: HD_Lab, HD_Literature - Descriptive Statistics

| Sample | N | Mean | StDev | SE Mean |
| --- | --- | --- | --- | --- |
| HD_Lab | 10 | 25.5499 | 0.1144 | 0.0362 |
| HD_Literature | 10 | 27.6010 | 0.2865 | 0.0906 |

Estimation for Paired Difference

| Mean | StDev | SE Mean | 95% CI for μ_difference |
| --- | --- | --- | --- |
| -2.0511 | 0.2559 | 0.0809 | (-2.2341, -1.8680) |

*µ_difference: mean of (HD_Lab - HD_Literature)*

Test

| Null hypothesis | H₀: μ_difference = 0 |
| --- | --- |
| Alternative hypothesis | H₁: μ_difference ≠ 0 |

| T-Value | P-Value |
| --- | --- |
| -25.35 | 0.000 |

**S2. Contact angle analysis**

***a.Contact angle of DI water on PET as a function of treatment time***


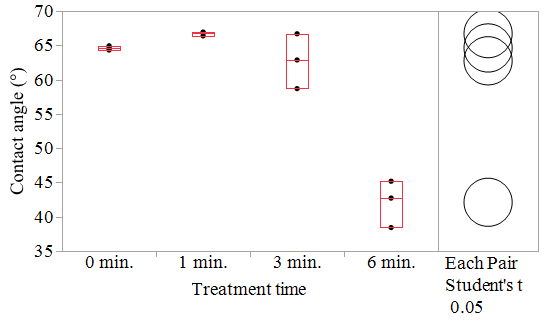

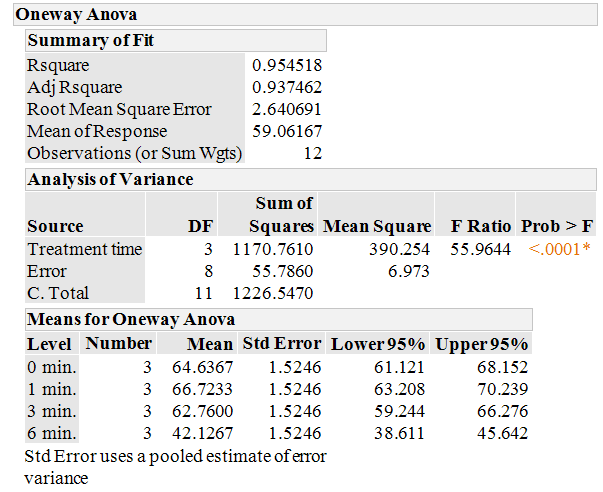


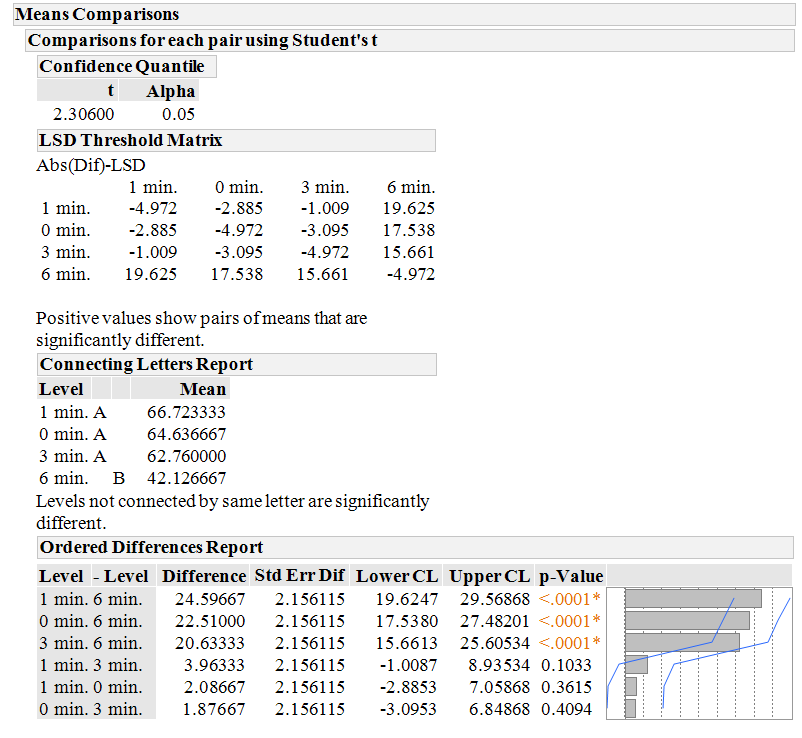


***b.Contact angle of MI on PET as a function of treatment time***


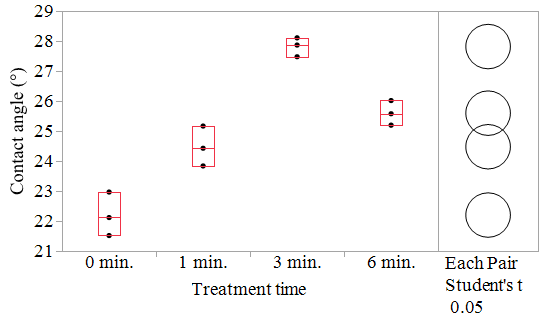

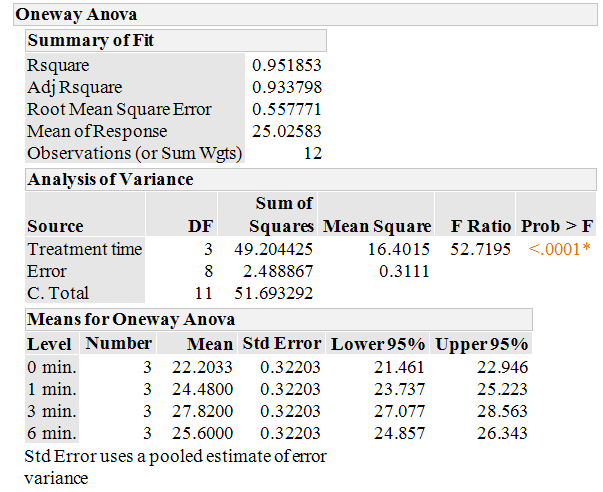


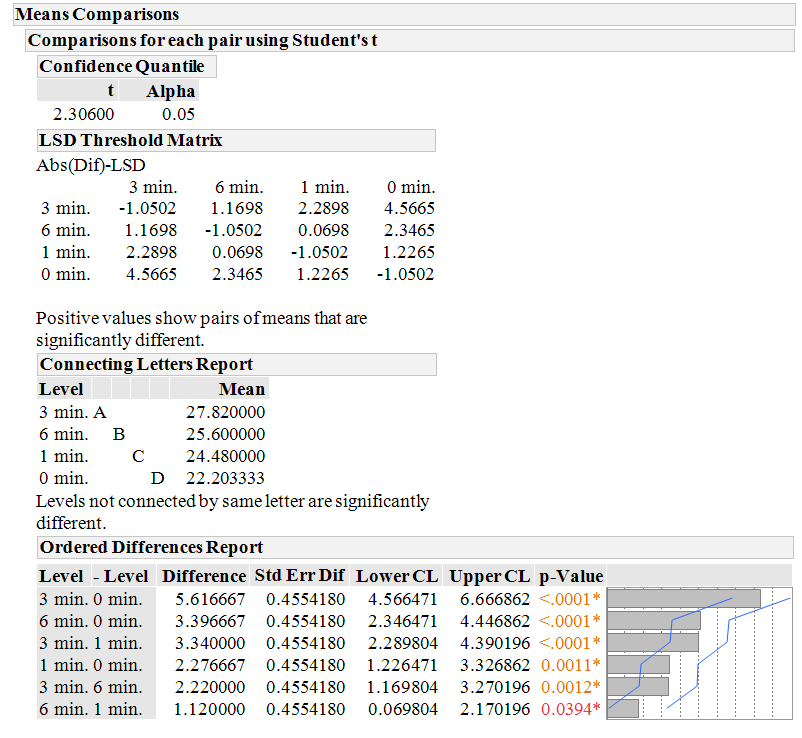


***c.Contact angle of HD on PET as a function of treatment time***


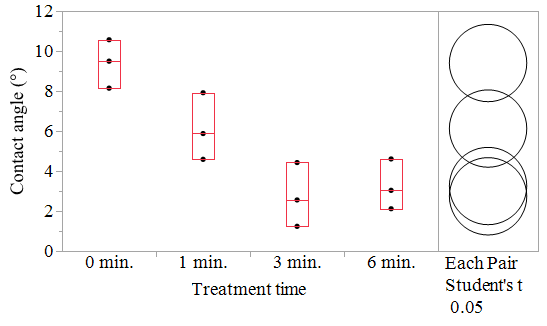

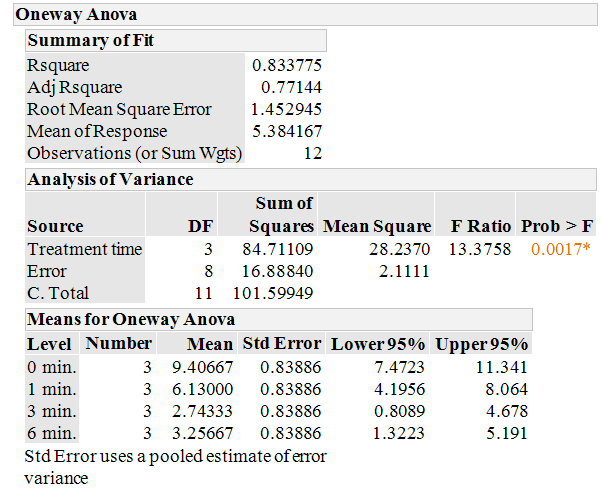


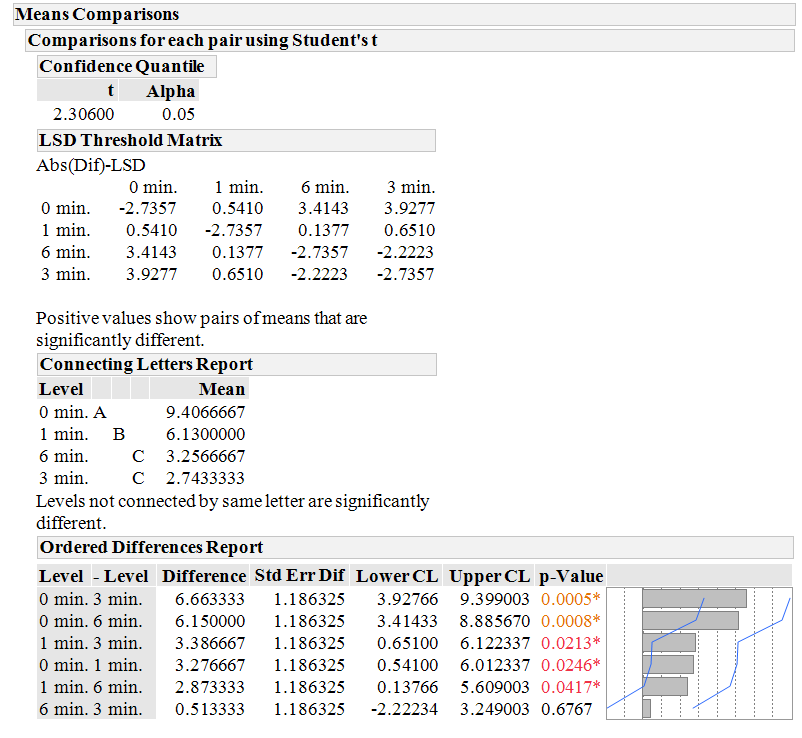


**S3. Owens-Wendt method: Paired-t test**

***The effect of liquid pairs on surface energy_experimental data (lab)***

Paired T-Test and CI: DI/MI_Lab_0, MI/HD_Lab_0 - Descriptive Statistics

| Sample | N | Mean | StDev | SE Mean |
| --- | --- | --- | --- | --- |
| DI/MI_Lab_0 | 3 | 47.54 | 2.85 | 1.65 |
| MI/HD_Lab_0 | 3 | 88.32 | 8.63 | 4.98 |

Estimation for Paired Difference

| Mean | StDev | SE Mean | 95% CI for μ_difference |
| --- | --- | --- | --- |
| -40.78 | 5.78 | 3.34 | (-55.14, -26.42) |

*µ_difference: mean of (DI/MI_Lab_0 - MI/HD_Lab_0)*

Test

| Null hypothesis | H₀: μ_difference = 0 |
| --- | --- |
| Alternative hypothesis | H₁: μ_difference ≠ 0 |

| T-Value | P-Value |
| --- | --- |
| -12.22 | 0.007* |

Paired T-Test and CI: DI/MI_Lab_0, DI/HD_Lab_0 - Descriptive Statistics

| Sample | N | Mean | StDev | SE Mean |
| --- | --- | --- | --- | --- |
| DI/MI_Lab_0 | 3 | 47.54 | 2.85 | 1.65 |
| DI/HD_Lab_0 | 3 | 40.49 | 1.45 | 0.84 |

Estimation for Paired Difference

| Mean | StDev | SE Mean | 95% CI for μ_difference |
| --- | --- | --- | --- |
| 7.050 | 1.400 | 0.808 | (3.572, 10.528) |

*µ_difference: mean of (DI/MI_Lab_0 - DI/HD_Lab_0)*

Test

| Null hypothesis | H₀: μ_difference = 0 |
| --- | --- |
| Alternative hypothesis | H₁: μ_difference ≠ 0 |

| T-Value | P-Value |
| --- | --- |
| 8.72 | 0.013* |

Paired T-Test and CI: DI/MI_Lab_1, MI/HD_Lab_1 - Descriptive Statistics

| Sample | N | Mean | StDev | SE Mean |
| --- | --- | --- | --- | --- |
| DI/MI_Lab_1 | 3 | 46.30 | 2.76 | 1.59 |
| MI/HD_Lab_1 | 3 | 83.27 | 8.13 | 4.69 |

Estimation for Paired Difference

| Mean | StDev | SE Mean | 95% CI for μ_difference |
| --- | --- | --- | --- |
| -36.97 | 5.37 | 3.10 | (-50.31, -23.63) |

*µ_difference: mean of (DI/MI_Lab_1 - MI/HD_Lab_1)*

Test

| Null hypothesis | H₀: μ_difference = 0 |
| --- | --- |
| Alternative hypothesis | H₁: μ_difference ≠ 0 |

| T-Value | P-Value |
| --- | --- |
| -11.92 | 0.007* |

Paired T-Test and CI: DI/MI_Lab_1, DI/HD_Lab_1 - Descriptive Statistics

| Sample | N | Mean | StDev | SE Mean |
| --- | --- | --- | --- | --- |
| DI/MI_Lab_1 | 3 | 46.30 | 2.76 | 1.59 |
| DI/HD_Lab_1 | 3 | 39.25 | 1.19 | 0.69 |

Estimation for Paired Difference

| Mean | StDev | SE Mean | 95% CI for μ_difference |
| --- | --- | --- | --- |
| 7.050 | 1.570 | 0.906 | (3.150, 10.950) |

*µ_difference: mean of (DI/MI_Lab_1 - DI/HD_Lab_1)*

Test

| Null hypothesis | H₀: μ_difference = 0 |
| --- | --- |
| Alternative hypothesis | H₁: μ_difference ≠ 0 |

| T-Value | P-Value |
| --- | --- |
| 7.78 | 0.016* |

Paired T-Test and CI: DI/MI_Lab_3, MI/HD_Lab_3 - Descriptive Statistics

| Sample | N | Mean | StDev | SE Mean |
| --- | --- | --- | --- | --- |
| DI/MI_Lab_3 | 3 | 46.83 | 3.91 | 2.26 |
| MI/HD_Lab_3 | 3 | 76.66 | 7.23 | 4.17 |

Estimation for Paired Difference

| Mean | StDev | SE Mean | 95% CI for μ_difference |
| --- | --- | --- | --- |
| -29.83 | 3.32 | 1.92 | (-38.08, -21.58) |

*µ_difference: mean of (DI/MI_Lab_3 - MI/HD_Lab_3)*

Test

| Null hypothesis | H₀: μ_difference = 0 |
| --- | --- |
| Alternative hypothesis | H₁: μ_difference ≠ 0 |

| T-Value | P-Value |
| --- | --- |
| -15.56 | 0.004* |

Paired T-Test and CI: DI/MI_Lab_3, DI/HD_Lab_3 - Descriptive Statistics

| Sample | N | Mean | StDev | SE Mean |
| --- | --- | --- | --- | --- |
| DI/MI_Lab_3 | 3 | 46.83 | 3.91 | 2.26 |
| DI/HD_Lab_3 | 3 | 41.75 | 1.93 | 1.11 |

Estimation for Paired Difference

| Mean | StDev | SE Mean | 95% CI for μ_difference |
| --- | --- | --- | --- |
| 5.08 | 1.98 | 1.14 | (0.16, 10.00) |

*µ_difference: mean of (DI/MI_Lab_3 - DI/HD_Lab_3)*

Test

| Null hypothesis | H₀: μ_difference = 0 |
| --- | --- |
| Alternative hypothesis | H₁: μ_difference ≠ 0 |

| T-Value | P-Value |
| --- | --- |
| 4.44 | 0.047* |

Paired T-Test and CI: DI/MI_Lab_6, MI/HD_Lab_6 - Descriptive Statistics

| Sample | N | Mean | StDev | SE Mean |
| --- | --- | --- | --- | --- |
| DI/MI_Lab_6 | 3 | 57.10 | 4.61 | 2.66 |
| MI/HD_Lab_6 | 3 | 80.76 | 7.58 | 4.38 |

Estimation for Paired Difference

| Mean | StDev | SE Mean | 95% CI for μ_difference |
| --- | --- | --- | --- |
| -23.66 | 2.97 | 1.71 | (-31.04, -16.28) |

*µ_difference: mean of (DI/MI_Lab_6 - MI/HD_Lab_6)*

Test

| Null hypothesis | H₀: μ_difference = 0 |
| --- | --- |
| Alternative hypothesis | H₁: μ_difference ≠ 0 |

| T-Value | P-Value |
| --- | --- |
| -13.80 | 0.005* |

Paired T-Test and CI: DI/MI_Lab_6, DI/HD_Lab_6 - Descriptive Statistics

| Sample | N | Mean | StDev | SE Mean |
| --- | --- | --- | --- | --- |
| DI/MI_Lab_6 | 3 | 57.10 | 4.61 | 2.66 |
| DI/HD_Lab_6 | 3 | 55.11 | 2.39 | 1.38 |

Estimation for Paired Difference

| Mean | StDev | SE Mean | 95% CI for μ_difference |
| --- | --- | --- | --- |
| 1.99 | 2.22 | 1.28 | (-3.52, 7.50) |

*µ_difference: mean of (DI/MI_Lab_6 - DI/HD_Lab_6)*

Test

| Null hypothesis | H₀: μ_difference = 0 |
| --- | --- |
| Alternative hypothesis | H₁: μ_difference ≠ 0 |

| T-Value | P-Value |
| --- | --- |
| 1.55 | 0.261 |

***The effect of liquid pairs on surface energy_Literature versus Experimental (lab)***

Paired T-Test and CI: DI/MI_Lab_0, DI/MI_Literature_0 – Descriptive Statistics

| Sample | N | Mean | StDev | SE Mean |
| --- | --- | --- | --- | --- |
| DI/MI_Lab_0 | 3 | 47.54 | 2.85 | 1.65 |
| DI/MI_Literature_0 | 3 | 49.80 | 1.32 | 0.76 |

Estimation for Paired Difference

| Mean | StDev | SE Mean | 95% CI for μ_difference |
| --- | --- | --- | --- |
| -2.260 | 1.530 | 0.883 | (-6.061, 1.541) |

*µ_difference: mean of (DI/MI_Lab_0 – DI/MI_Literature_0)*

Test

| Null hypothesis | H₀: μ_difference = 0 |
| --- | --- |
| Alternative hypothesis | H₁: μ_difference ≠ 0 |

| T-Value | P-Value |
| --- | --- |
| -2.56 | 0.125 |

Paired T-Test and CI: DI/MI_Lab_1, DI/MI_Literature_1 – Descriptive Statistics

| Sample | N | Mean | StDev | SE Mean |
| --- | --- | --- | --- | --- |
| DI/MI_Lab_1 | 3 | 46.30 | 2.76 | 1.59 |
| DI/MI_Literature_1 | 3 | 48.54 | 1.29 | 0.74 |

Estimation for Paired Difference

| Mean | StDev | SE Mean | 95% CI for μ_difference |
| --- | --- | --- | --- |
| -2.240 | 1.470 | 0.849 | (-5.892, 1.412) |

*µ_difference: mean of (DI/MI_Lab_1 – DI/MI_Literature_1)*

Test

| Null hypothesis | H₀: μ_difference = 0 |
| --- | --- |
| Alternative hypothesis | H₁: μ_difference ≠ 0 |

| T-Value | P-Value |
| --- | --- |
| -2.64 | 0.119 |

Paired T-Test and CI: DI/MI_Lab_3, DI/MI_Literature_3 – Descriptive Statistics

| Sample | N | Mean | StDev | SE Mean |
| --- | --- | --- | --- | --- |
| DI/MI_Lab_3 | 3 | 46.83 | 3.91 | 2.26 |
| DI/MI_Literature_3 | 3 | 48.94 | 2.35 | 1.36 |

Estimation for Paired Difference

| Mean | StDev | SE Mean | 95% CI for μ_difference |
| --- | --- | --- | --- |
| -2.110 | 1.560 | 0.901 | (-5.985, 1.765) |

*µ_difference: mean of (DI/MI_Lab_3 – DI/MI_Literature_3)*

Test

| Null hypothesis | H₀: μ_difference = 0 |
| --- | --- |
| Alternative hypothesis | H₁: μ_difference ≠ 0 |

| T-Value | P-Value |
| --- | --- |
| -2.34 | 0.144 |

Paired T-Test and CI: DI/MI_Lab_6, DI/MI_Literature_6 – Descriptive Statistics

| Sample | N | Mean | StDev | SE Mean |
| --- | --- | --- | --- | --- |
| DI/MI_Lab_6 | 3 | 57.10 | 4.61 | 2.66 |
| DI/MI_Literature_6 | 3 | 59.04 | 2.54 | 1.47 |

Estimation for Paired Difference

| Mean | StDev | SE Mean | 95% CI for μ_difference |
| --- | --- | --- | --- |
| -1.94 | 2.07 | 1.20 | (-7.08, 3.20) |

*µ_difference: mean of (DI/MI_Lab_6 - DI/MI_Literature_6)*

Test

| Null hypothesis | H₀: μ_difference = 0 |
| --- | --- |
| Alternative hypothesis | H₁: μ_difference ≠ 0 |
| T-Value | P-Value |
| -1.62 | 0.246 |

Paired T-Test and CI: MI/HD_Lab_0, MI/HD_Literature_0 - Descriptive Statistics

| Sample | N | Mean | StDev | SE Mean |
| --- | --- | --- | --- | --- |
| MI/HD_Lab_0 | 3 | 88.32 | 8.63 | 4.98 |
| MI/HD_Literature_0 | 3 | 89.72 | 8.10 | 4.68 |

Estimation for Paired Difference

| Mean | StDev | SE Mean | 95% CI for μ_difference |
| --- | --- | --- | --- |
| -1.400 | 0.530 | 0.306 | (-2.717, -0.083) |

*µ_difference: mean of (MI/HD_Lab_0 - MI/HD_Literature_0)*

Test

| Null hypothesis | H₀: μ_difference = 0 |
| --- | --- |
| Alternative hypothesis | H₁: μ_difference ≠ 0 |

| T-Value | P-Value |
| --- | --- |
| -4.58 | 0.045* |

Paired T-Test and CI: MI/HD_Lab_1, MI/HD_Literature_1 - Descriptive Statistics

| Sample | N | Mean | StDev | SE Mean |
| --- | --- | --- | --- | --- |
| MI/HD_Lab_1 | 3 | 83.27 | 8.13 | 4.69 |
| MI/HD_Literature_1 | 3 | 84.55 | 7.64 | 4.41 |

Estimation for Paired Difference

| Mean | StDev | SE Mean | 95% CI for μ_difference |
| --- | --- | --- | --- |
| -1.280 | 0.490 | 0.283 | (-2.497, -0.063) |

*µ_difference: mean of (MI/HD_Lab_1 - MI/HD_Literature_1)*

Test

| Null hypothesis | H₀: μ_difference = 0 |
| --- | --- |
| Alternative hypothesis | H₁: μ_difference ≠ 0 |

| T-Value | P-Value |
| --- | --- |
| -4.52 | 0.046* |

Paired T-Test and CI: MI/HD_Lab_3, MI/HD_Literature_3 - Descriptive Statistics

| Sample | N | Mean | StDev | SE Mean |
| --- | --- | --- | --- | --- |
| MI/HD_Lab_3 | 3 | 76.66 | 7.23 | 4.17 |
| MI/HD_Literature_3 | 3 | 77.81 | 6.81 | 3.93 |

Estimation for Paired Difference

| Mean | StDev | SE Mean | 95% CI for μ_difference |
| --- | --- | --- | --- |
| -1.150 | 0.420 | 0.242 | (-2.193, -0.107) |

*µ_difference: mean of (MI/HD_Lab_3 - MI/HD_Literature_3)*

Test

| Null hypothesis | H₀: μ_difference = 0 |
| --- | --- |
| Alternative hypothesis | H₁: μ_difference ≠ 0 |

| T-Value | P-Value |
| --- | --- |
| -4.74 | 0.042* |

Paired T-Test and CI: MI/HD_Lab_6, MI/HD_Literature_6 - Descriptive Statistics

| Sample | N | Mean | StDev | SE Mean |
| --- | --- | --- | --- | --- |
| MI/HD_Lab_6 | 3 | 80.76 | 7.58 | 4.38 |
| MI/HD_Literature_6 | 3 | 81.99 | 7.11 | 4.10 |

Estimation for Paired Difference

| Mean | StDev | SE Mean | 95% CI for μ_difference |
| --- | --- | --- | --- |
| -1.230 | 0.470 | 0.271 | (-2.398, -0.062) |

*µ_difference: mean of (MI/HD_Lab_6 - MI/HD_Literature_6)*

Test

| Null hypothesis | H₀: μ_difference = 0 |
| --- | --- |
| Alternative hypothesis | H₁: μ_difference ≠ 0 |

| T-Value | P-Value |
| --- | --- |
| -4.53 | 0.045* |

Paired T-Test and CI: DI/HD_Lab_0, DI/HD_Literature_0 - Descriptive Statistics

| Sample | N | Mean | StDev | SE Mean |
| --- | --- | --- | --- | --- |
| DI/HD_Lab_0 | 3 | 40.490 | 1.450 | 0.837 |
| DI/HD_Literature_0 | 3 | 42.200 | 1.500 | 0.866 |

Estimation for Paired Difference

| Mean | StDev | SE Mean | 95% CI for μ_difference |
| --- | --- | --- | --- |
| -1.7100 | 0.0500 | 0.0289 | (-1.8342, -1.5858) |

*µ_difference: mean of (DI/HD_Lab_0 - DI/HD_Literature_0)*

Test

| Null hypothesis | H₀: μ_difference = 0 |
| --- | --- |
| Alternative hypothesis | H₁: μ_difference ≠ 0 |

| T-Value | P-Value |
| --- | --- |
| -59.24 | 0.000* |

Paired T-Test and CI: DI/HD_Lab_1, DI/HD_Literature_1 - Descriptive Statistics

| Sample | N | Mean | StDev | SE Mean |
| --- | --- | --- | --- | --- |
| DI/HD_Lab_1 | 3 | 39.250 | 1.190 | 0.687 |
| DI/HD_Literature_1 | 3 | 40.990 | 1.250 | 0.722 |

Estimation for Paired Difference

| Mean | StDev | SE Mean | 95% CI for μ_difference |
| --- | --- | --- | --- |
| -1.7400 | 0.0600 | 0.0346 | (-1.8890, -1.5910) |

*µ_difference: mean of (DI/HD_Lab_1 - DI/HD_Literature_1)*

Test

| Null hypothesis | H₀: μ_difference = 0 |
| --- | --- |
| Alternative hypothesis | H₁: μ_difference ≠ 0 |

| T-Value | P-Value |
| --- | --- |
| -50.23 | 0.000* |

Paired T-Test and CI: DI/HD_Lab_3, DI/HD_Literature_3 - Descriptive Statistics

| Sample | N | Mean | StDev | SE Mean |
| --- | --- | --- | --- | --- |
| DI/HD_Lab_3 | 3 | 41.75 | 1.93 | 1.11 |
| DI/HD_Literature_3 | 3 | 43.48 | 1.93 | 1.11 |

Estimation for Paired Difference

| Mean | StDev | SE Mean | 95% CI for μ_difference |
| --- | --- | --- | --- |
| -1.730 | 0.000 | 0.000 | (-1.730, -1.730) |

*µ_difference: mean of (DI/HD_Lab_3 - DI/HD_Literature_3)*

Test

| Null hypothesis | H₀: μ_difference = 0 |
| --- | --- |
| Alternative hypothesis | H₁: μ_difference ≠ 0 |

| T-Value | P-Value |
| --- | --- |
| * | * * NOTE * All values in column are identical. |

Paired T-Test and CI: DI/HD_Lab_6, DI/HD_Literature_6 - Descriptive Statistics

| Sample | N | Mean | StDev | SE Mean |
| --- | --- | --- | --- | --- |
| DI/HD_Lab_6 | 3 | 55.11 | 2.39 | 1.38 |
| DI/HD_Literature_6 | 3 | 56.80 | 2.14 | 1.24 |

Estimation for Paired Difference

| Mean | StDev | SE Mean | 95% CI for μ_difference |
| --- | --- | --- | --- |
| -1.690 | 0.250 | 0.144 | (-2.311, -1.069) |

*µ_difference: mean of (DI/HD_Lab_6 - DI/HD_Literature_6)*

Test

| Null hypothesis | H₀: μ_difference = 0 |
| --- | --- |
| Alternative hypothesis | H₁: μ_difference ≠ 0 |

| T-Value | P-Value |
| --- | --- |
| -11.71 | 0.007* |
